# Supplementary material for: Identification of photoperiod-induced specific miRNAs in the adrenal glands of Sunite sheep (Ovis aries)
Source: Front Vet Sci. 2022 Jul 22;9:888207. doi: 10.3389/fvets.2022.888207 (PMC9354845; doi:10.3389/fvets.2022.888207)
Supplement: Supplementary file 1 [file Data_Sheet_1.ZIP › Supplementary file/miRDeep2 file.zip!/miRDeep2 file/Know_Structure/SPLP42A1/oar-mir-10b.pdf]

miRBase precursor	: oar-mir-10b
Total read count	: 574803
oar-miR-10b read count	: 572337
remaining reads	: 2466

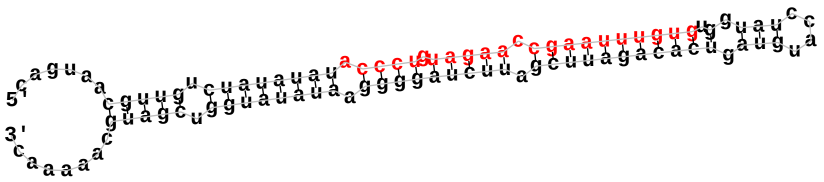

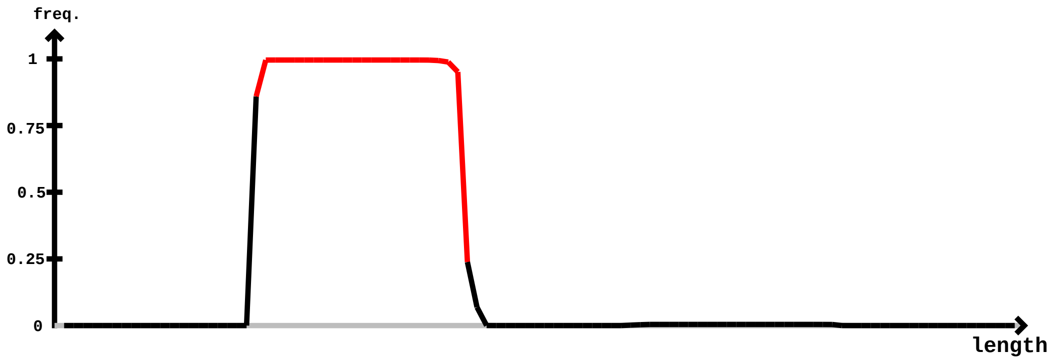

oar-miR-10b

5'- caguaacguugucuauauauacccuguagaaccgaauuugugugguauccauguagucacagauucgauucuaggggaauauauggucgaugcaaaaac -3'	exp		
......(((((.((((((((.((((.(((((.((((((((((.(.(((....))).))))))))))))).)))))))))).)))))))).)))))).......	reads	mm	sample
..................auacccuguagaaccgaauuug...	1	0	seq
..................auacccuguagaaccgaauuugu..	11	0	seq
..................auacccuguagaaccgaauuugug...	1	0	seq
..................auacccuguagaaccgaauuugugu..	1	0	seq
...................uacccuguagaaccgaa...	7	0	seq
...................uacccuguagaaccgaau..	89	0	seq
...................uacccuguagaaccgaauu...	976	0	seq
...................uacccuguagaaccgaauuu..	2830	0	seq
...................uacccuguagaaccgaauuug...	19592	0	seq
...................uacccuguagaaccgaauuugu..	383994	0	seq
...................uacccuguagaaccgaauuugug...	70957	0	seq
...................uacccuguagaaccgaauuugugu..	15388	0	seq
...................uacccuguagaaccgaauuugugug...	26	0	seq
...................uacccuguagaaccgaauuuguguggg..	6	0	seq
...................uacccuguagaaccgaauuugugugguu...	2	0	seq
...................uacccuguagaaccgaauuugugugguaa..	1	0	seq
....................acccuguagaaccgaau..	3	0	seq
....................acccuguagaaccgaauu...	88	0	seq
....................acccuguagaaccgaauuu..	369	0	seq
....................acccuguagaaccgaauuug...	1674	0	seq
....................acccuguagaaccgaauuugu..	25739	0	seq
....................acccuguagaaccgaauuugug...	26572	0	seq
....................acccuguagaaccgaauuugugu..	23848	0	seq
....................acccuguagaaccgaauuugugug...	92	0	seq
....................acccuguagaaccgaauuuguguggg..	6	0	seq
.....................cccuguagaaccgaauuugu..	3	0	seq
.....................cccuguagaaccgaauuugug...	9	0	seq
.....................cccuguagaaccgaauuugugu..	26	0	seq
......................ccuguagaaccgaauuugu..	4	0	seq
......................ccuguagaaccgaauuugug...	6	0	seq
......................ccuguagaaccgaauuugugu..	5	0	seq
......................ccuguagaaccgaauuugugug...	1	0	seq
.......................cuguagaaccgaauuugu..	3	0	seq
.......................cuguagaaccgaauuugug...	4	0	seq
.......................cuguagaaccgaauuugugu..	3	0	seq

oar-miR-10b

```
caguaacguugucuauauauacccuguagaaccgaauuugugugguauccauguagucacagauucgauucuaggggaauauauggucgaugcaaaaac
...................................................uguagucacagauucgauucuaggggaaua..................   1     0    seq
.........................................................cacagauucgauucuagggg......................   1     0    seq
.........................................................cacagauucgauucuagggga.....................   6     0    seq
.........................................................cacagauucgauucuaggggaa....................   1     0    seq
.........................................................cacagauucgauucuaggggaau...................   8     0    seq
..........................................................acagauucgauucuagg........................   1     0    seq
..........................................................acagauucgauucuagggg......................   3     0    seq
..........................................................acagauucgauucuagggga.....................   27    0    seq
..........................................................acagauucgauucuaggggaa....................   40    0    seq
..........................................................acagauucgauucuaggggaau...................   860   0    seq
..........................................................acagauucgauucuaggggaaua..................   2     0    seq
...........................................................cagauucgauucuagggg......................   3     0    seq
...........................................................cagauucgauucuagggga.....................   19    0    seq
...........................................................cagauucgauucuaggggaa....................   23    0    seq
...........................................................cagauucgauucuaggggaau...................   843   0    seq
...........................................................cagauucgauucuaggggaaua..................   8     0    seq
...........................................................cagauucgauucuaggggaauau.................   6     0    seq
............................................................agauucgauucuagggga.....................   8     0    seq
............................................................agauucgauucuaggggaa....................   7     0    seq
............................................................agauucgauucuaggggaau...................   550   0    seq
............................................................agauucgauucuaggggaaua..................   27    0    seq
............................................................agauucgauucuaggggaauau.................   21    0    seq
.............................................................gauucgauucuaggggaauau.................   1     0    seq
```
